# Discovery and mechanistic profiling of novel kokumi peptides from Wuding chicken soup

**DOI:** 10.1016/j.fochx.2025.103171

**Published:** 2025-10-14

**Authors:** Chunfang Yang, Yanfei Du, Guiying Wang, Jia Liu, Jiayan Tan, Shuai Tang, Yuan Liu, Hongbin Pan, Guozhou Liao

**Affiliations:** a College of Food Science and Technology, Yunnan Agricultural University, Kunming 650201, China; b Livestock Product Processing and Engineering Technology Research Center of Yunnan Province, Yunnan Agricultural University, Kunming 650201, China; c School of Food Science and Engineering, Ningxia University, Yinchuan 750021, China; d College of Animal Science and Technology, Yunnan Agricultural University, Kunming 650201, China

**Keywords:** Kokumi peptides, Hydrophobic interaction, Hydrogen bonding, CaSR, Wuding chicken

## Abstract

This study aims to identify potential kokumi peptides in Wuding chicken soup. To enrich oligopeptides, the preparation process was optimized, and a combined approach using ultrafiltration and gel filtration chromatography was employed for separation and purification. Sensory analysis was then integrated to screen and identify kokumi peptides. And an in-depth study was conducted on the binding mechanism of peptides and calcium-sensing receptors (CaSR). Fifteen kokumi peptides were identified, of which four (LPVPAFN, SAAPAAPVPA, VPAFNVING, and AGFAGDDAPR) were selected for detailed analysis. The primary active sites mediating the interaction between these peptides and the CaSR were identified as Arg 66, Ser 303, Ser 147, Glu 297, and Arg 69, with hydrophobic interactions and hydrogen bonding serving as the predominant binding forces. As the first discovery in Wuding chicken soup, this study establishes a methodological framework for kokumi peptide screening in local chicken varieties and advances structure-activity relationship insights for future applications.

## Highlights

• Four novel kokumi peptides were found in Wuding chicken soup.
• Kokumi peptides mainly bind to the A chain of the receptor CaSR.
• Arg 66, Ser 303, Ser 147, Glu 297, and Arg 69 were the key amino acid residues.
• Hydrogen bonding and hydrophobic interaction were the main binding forces.

## Graphical abstract

**Figure:**
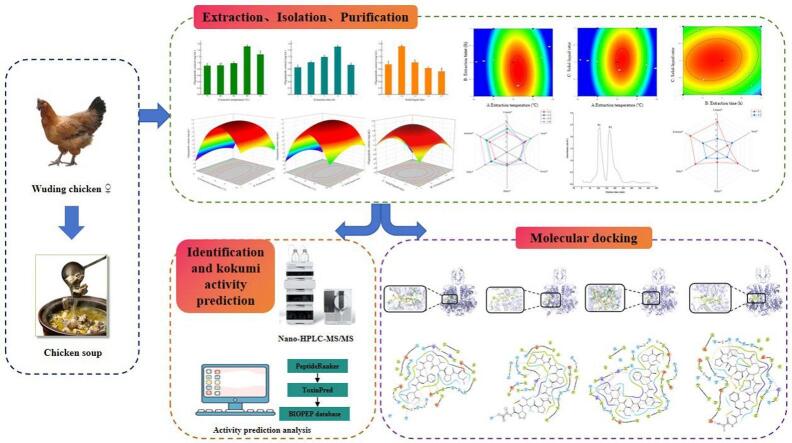

## 1 Introduction

Many studies have been conducted on kokumi flavor components, which are thought to be sensory substances that cause unique taste perception different from five basic tastes (sour, sweet, bitter, salty, and umami) (Ueda et al., 1994). With the improvement of people's quality of life, a single taste is no longer sufficient to meet everyone's needs. A single taste cannot fully convey the characteristics of certain substances, such as thickness, continuity, complexity, balance and roundness (Yamamoto & Inui-Yamamoto, 2023). Japanese researchers first proposed the concept of “kokumi taste” when examining garlic water extract. This taste can give people a thickness, continuous mouthfulness, and other sensory stimuli, allowing them to feel the thickness and durability of the overall taste. The substance that can cause this feeling is named “kokumi” (Ueda et al., 1990). Kokumi compounds are currently receiving more and more attention in the fields of research and the food business. They serve as flavor enhancers, adding depth and savor to a variety of food items, such as meat products, soups, sauces, and fermented foods (Kim et al., 2022).

Five basic taste receptors are not the same as kokumi taste receptors. G protein-coupled receptors moderate the sweet, bitter, and umami flavors of the five fundamental tastes that are mediated by taste buds, while specific membrane channels regulate the sour and salty senses. However, the kokumi taste receptor is a calcium- sensitive receptor (CaSR) (Beauchamp, 2019; Spaggiari et al., 2020). The basic taste stimulate the taste receptor cells to respond and transmit them to the brain in the form of electrical signals, ultimately producing taste. In contrast, the taste of kokumi mainly stimulates CaSR, which increases calcium ion flux in cells and triggers a response. Generally speaking, kokumi substances are tasteless and even have some special tastes, such as glutathione. Polypeptides with different molecular weights present different tastes. Sentandreu et al. (2010) and M-Concepción Aristoy et al. (1995) realized that peptides with a molecular weights less than 3000 Da could bring various tastes to Spanish dry-cured ham. Among them, peptides with molecular weights between 1500 and 1700 Da had a umami flavor, while those with molecular weights between 1700 and 1800 Da had a bitter taste. However, according to Liang et al. (2022), oligopeptides with molecular weights below 1000 Da are the main flavoring agents in yeast extracts, chicken soup and Jinhua ham. Rhyu and Kim (2011) also discovered that peptides with molecular weights between 500 and 1000 Da have the strongest flavor effect when studying soybean paste. In Agaricus bisporus, Feng et al. (2024) found peptides with molecular weights ranging from 200 to 1000 Da have the strongest thickness flavor. Kokumi peptides can be found in many different foods, including by-products of enzyme-modified butter (Yang et al., 2022), chicken protein hydrolysate (Zhang, Ma, et al., 2019), and yeast extract (Lao et al., 2024). Many undeveloped kokumi peptides still need further exploration and discovery.

Wuding chicken is an indigenous poultry breed predominantly found in Wuding County, Chuxiong Yi Ethnic Autonomous Prefecture, and is a geographically protected genetic resource in Yunnan's agricultural ecosystem. Characterized by exceptional body mass and organoleptic properties distinct from common broiler varieties, this avian species has gained substantial consumer preference in regional markets (Yu et al., 2020). Research on Wuding chicken involves multiple aspects, including processing techniques, metabolites, the composition of fatty acids, low molecular weight substances, volatile taste substances, and qualitative characteristics (Xiao et al., 2019; Xiao, & C., Luo, Y, T., Wang, G, Y., Ge, C, R., Zhou, G, H., Zhang, W, A. & Liao, G, Z., 2018). Although the quality of Wuding chicken have been thoroughly studied by researchers, no publications on the kokumi peptides found in Wuding chicken have been made. Presently, two kokumi peptides from Agaricus bisporus, EWVPVTK and EYPPLGR, have been discovered by Feng et al. (2024), while Lao et al. (2024) isolated kokumi peptides IQGFK and EDFFVR from yeast extract. Additionally, Yamasaki and Maekawa (1978) obtained the flavor-enhancing octapeptide KGDEESLA by hydrolyzing beef with papain. While the isolation of kokumi-active polypeptides has been extensively documented across diverse protein matrices, including marine organisms and legumes, the systematic characterization of these flavor-enhancing peptides in avian-derived thermal hydrolysis products remains conspicuously understudied. This knowledge gap highlights the scientific novelty of investigating chicken soup as a potential source of kokumi substances. Therefore, Wuding chicken was selected as research object in this study to efficiently detect and screen kokumi peptides in chicken soup, aiming to provide scientific basis for further exploration in this field.

In order to extract kokumi peptide from Wuding chicken soup to the maximum extent, traditional methods such as ultrafiltration (UF) and gel filtration chromatography (GFC) were employed for isolation and purification. The most potent kokumi components were identified through sensory evaluation. Sequence identification was performed using nanoscale liquid chromatography tandem mass spectrometry (Nano-HPLC-MS/MS), and kokumi peptides were screened based on peptidomics. Molecular docking was utilized to study the binding sites and interaction forces between kokumi peptides and their receptors. This research provides a foundation for finding and evaluating kokumi peptides in Wuding chicken soup and aids in the comprehension of the connection between these peptides' structure and action. The findings offer a theoretical basis for developing compound flavor peptide condiments, and have significant theoretical and practical value for promoting the deep processing and industrial development of Yunnan native chicken, thus enriching the theoretical framework of kokumi peptides.

## 2 Materials and methods

### 2.1 Materials and chemicals

Forty 300-day-old female Wuding chickens were uniformly slaughtered by Wuding Zhenji Agricultural Science and Technology Development Co., Ltd. (Yunnan, China), sealed in sterile bags, and brought to the lab between 0 and 4 °C. Glutathione (GSH), sucrose, citric acid, sodium chloride, monosodium glutamate (MSG), and quinine were food-grade provided by Kunming Renke Trading Co., Ltd. (Yunnan, China). All other solvents used are of analytical grade and chromatographic grade, and are prepared with ultrapure water.

### 2.2 Optimization of peptide extraction conditions for kokumi in Wuding chicken soup

A 100 g sample of chicken breast was weighed. A systematic study was conducted by varying the extraction temperature (75 °C, 80 °C, 85 °C, 90 °C, 95 °C), the amount of extraction time (1 h, 2 h, 3 h, 4 h, 5 h), and solid-liquid ratio (1:1, 1:2, 1:3, 1:4, 1:5). The optimal conditions for each factor were determined through oligopeptide content, utilizing the principles of the Box-Behnken design and Design-Expert 13 software. Three factors were studied: extraction temperature, extraction time, and solid-liquid ratio, with each factor having three coded levels of −1, 0, and 1 (Table S1). A total of 17 experimental runs, including five center points, were conducted according to the experimental design.

Based on the test results of single-factor and response surface, the chicken soup was centrifuged at 4200 r/min for 25 min at 4 °C to eliminate the dissolved solids. The supernatant was then collected and kept at −80 °C for additional separation and purification.

### 2.3 Isolation and purification of peptides

The aforementioned supernatant was separated and purified using an ultrafiltration membrane (Mw = 1, 3, 5 kDa) and gel filtration chromatography (Sephadex G-15, UV = 220 nm) in that order, and the components with the kokumi taste were screened (Deng et al., 2021). First, various ultrafiltration membranes were used to separate the chicken soup supernatant. Following lyophilization of the separated components, the strongest kokumi components were reserved based on sensory analysis. GFC separation was the next stage. Following UF, the sample volume was set at 2 mL and filtered through a 0.45 μm aqueous membrane. The group with the highest kokumi taste was then diluted to a solution of 20 mg/mL. After separating the samples in GFC, an automatic part collector then repeatedly collected the peaks of these components and freeze-dried them for usage. Lastly, for final identification, the ingredients with the strongest kokumi taste were filtered through sensory evaluation.

### 2.4 Identification of peptides

Peptide identification was performed using the Nano-HPLC-MS/MS method, based on previous studies by the research group, with some adjustments (Jia et al., 2024). The specific method was evaluated using an Orbitrap Q Exactive Plus mass spectrometer coupled to an EASY-nanoLC 1200 system (Thermo Fisher Scientific, MA, USA) after being redissolved in solvent A (0.1 % formic acid in water). A 25 cm analytical column (75 μm inner diameter, 1.9 μm resin) was loaded with 1 μL of the peptide sample. The sample was separated using a 60-min gradient, beginning at 2 % buffer B (80 % ACN with 0.1 % FA) and increasing progressively to 32 % in 47 min, 100 % in 1 min, and remaining there for 12 min. The column temperature was kept at 40 °C while the flow rate was kept at 300 nL/min. Two kV was used as the electrospray voltage. The mass spectrometer automatically alternated between MS and MS/MS modes while operating in data-dependent acquisition (DDA) mode. Full scan MS spectra (*m*/*z* 200–2000) were surveyed using the Orbitrap at a resolution of 70,000. The maximum injection time is 50 ms, and the automatic gain control (AGC) target is 3e6. Higher-energy collision dissociation (HCD) was then used to select the precursor ions into the collision cell for fragmentation; the normalized collection energy was 28 %. The dynamic exclusion was set at 30 s, the maximum injection duration was 45 ms, the automatic gain control (AGC) target was 1e5, and the MS/MS resolution was set at 17500.

### 2.5 Kokumi activity prediction of peptides

The ToxinPred (https://webs.iiitd.edu.in/raghava/toxinpred/index.html) was used to predict toxic peptides, while BIOPEP-UWM database (https://www.uwm.edu.pl/ biochemia/index.php/pl/biopep) was used to predict the activity of peptide taste and investigate the frequency of bioactive fragments in target peptides (Zhang et al., 2022). Then, PeptideRanker (http://distilldeep.ucd.ie//PeptideRanker/) was used to predict the peptide activity (Wang et al., 2023). Finally, in comparison with the kokumi database in the Flavor Database (https://mffi.sjtu.edu.cn/database), the sequences that the database did not record were kept (Guo et al., 2024).

### 2.6 Molecular docking of kokumi peptides

The RCSB Protein Data Bank (https://www.rcsb.org/) provided the crystal structure of the active form of the extracellular domain of the human calcium-sensing receptor (CaSR) (PDB ID: 5K5S, resolution: 2.6 Å), which we used in this investigation (Geng et al., 2016). According to Geng et al. (2016), the Venus Flytrap (VFT) domain, the cysteine-rich domain, and the seven-helix transmembrane region are the three major structural domains of the 5K5S structure, which is made up of two chains (A and B) that form a disulfide-linked homodimer. It is noteworthy that kokumi peptides, which cause kokumi taste reactions, have been found to bind to the VFT domain (Dellafiora et al., 2022).

Using PyMOL software, the 5K5S structure was preprocessed by eliminating water molecules and ligands (PO^4+^, Ca^2+^, NAG) before molecular docking study. For structural modeling, the model was built using AlphaFold v2.3 (Jumper et al., 2021) through Docker image, and all computations were performed on the local Ubuntu 18.04 server. The modeling process utilized the “multimer” artificial intelligence model and integrated all genetic databases of CASP14. Following model generation, the Amber force field was applied for protein relaxation in OpenMM, and the software output's greatest plddt confidence score was used to choose the final experimental model.

### 2.7 Taste dilution analysis

Following the analytical approach described by Chang et al. (2023), Specifically, both U1 fraction and gel-filtration chromatography isolates were reconstituted at 5 mg/mL in phosphate buffer, followed by systematic binary dilutions. Sensory stimulation sequences were administered to panelists in decreasing concentration gradients, with 5 mL aliquots per dilution level ensuring standardized stimulus delivery. The taste dilution (TD) value was determined as the dilution ratio at which the evaluator could just distinguish the taste difference between the sample and two blank controls. Throughout the evaluation process, sensory assessors were instructed to avoid discussion or communication, and the average of three independent replicate tests was used to get the final TD value.

### 2.8 Sensory evaluation

A modified framework for sensory evaluation was used, incorporating stringent panelist selection criteria (Song et al., 2023). The sensory experiments of Yunnan Agricultural University do not require ethical approval from the Human Ethics Committee. Furthermore, our institution does not have a systematic documentation procedure for sensory research. The evaluators involved in sensory evaluation confirm that their rights and privacy can be protected without the need for ethical approval. Every participant signed the informed consent form, attesting to their knowledge of the experiment's possible dangers. Every participant gave their consent to participate in the study and was able to leave at any moment. Twenty qualified assessors, aged 20 to 25, who were selected from Yunnan Agricultural University's Food Science Department, made up the cohort. Every team member has worked in sensory evaluation for at least a year. The team members had no olfactory or taste impairments and were in good condition. After finishing the sensory training in compliance with the National Standard method (GB/T 16291.1–2012), participants can correctly recognize kokumi flavors and the five basic flavors—sour, sweet, bitter, salty, and umami. 0.08 % citric acid, 1 % sucrose, 0.01 % quinine sulfate, 0.35 % sodium chloride, and 0.35 % monosodium glutamate were used as evaluation standards for acidic, sweet, bitter, salty and umami tastes, and the samples were dissolved in deionized water. Furthermore, the standard for kokumi was 1 % glutathione in a model chicken broth solution. Panelists were instructed to sip the sample, allow it to swirl in their mouths for a short while, then expectorate during the sensory tests, which were held at (23 ± 2) °C. The panelists were instructed to rinse their mouths with 100 mL of water in between testing two distinct samples in order to prevent fatigue and carryover effects. Additionally, one hour before to the sensory evaluation, no food, beverages, or tobacco were permitted. Commercial Wuding chicken that had been cooked with a material-liquid ratio of 1:2 (chicken:water) was used to make the model chicken broth. The broth was filtered for later use after the froth and fat were skimmed off after an hour of steaming.

### 2.9 Statistical analysis

Software from Origin 2018, Microsoft Office Excel 2021, and SPSS 25.0 were used for analyzing all of the data. The significance criterion for the one-way ANOVA analysis was *P* < 0.05. The model of the thick peptide is constructed and optimized using ChemOffice 2014. AlphaFold v2.3, PyMOL 1.7, and Schrodinger software were used to visualize the structure and analyze the interaction force of the obtained thick peptide-thick receptor CaSR complex.

## 3 Results and discussion

### 3.1 Process optimization of single-factor and response surface extraction conditions for kokumi peptide in Wuding chicken soup

**Fig. 1:**
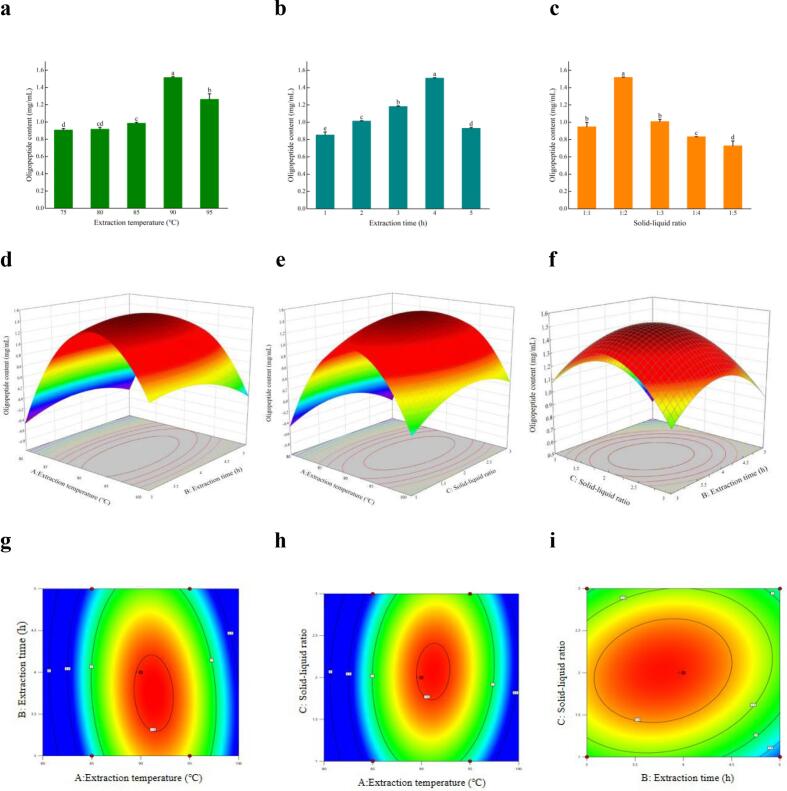
**Analysis results of single factor experiment.** (a) Extraction temperature; (b) Extraction time; (c) Solid-liquid ratio. Surface and contour plots of (d, g) extraction temperature and extraction time; (e, h) extraction temperature and solid-liquid ratio, and (f, i) extraction time and solid-liquid ratio.

By using the trace biuret method and taking the content of oligopeptides as the ordinate, the standard curve was obtained as: y = 0.719×- 0.0082, R^2^ = 0.9991. As shown in Fig. 1a, when the ratio of material to liquid and the extraction time are held constant, the oligopeptide content at 90 °C is significantly higher than at other extraction temperatures. As the cooking temperature increases, the oligopeptide content initially rises and then decreases. Although low-temperature treatment can achieve sterilization and cooking, it may not fully dissolve the soluble small-molecule polypeptides in the chicken soup, leaving more flavor substances in the chicken meat. When the temperature exceeds 90 °C, the oligopeptide content decreases, possibly because high temperatures damage the chicken's tissue structure, thereby reducing the dissolution of soluble small-molecule polypeptides. As shown in Fig. 1b, when the extraction temperature and the material-to-liquid ratio are held constant, different extraction time significantly affect the oligopeptide content (*P* < 0.05). The oligopeptide content reaches its maximum value at a extraction time of 4 h. With prolonged boiling, the oligopeptide content initially increases and then decreases. This trend may be due to the soluble substances in the soup reaching their maximum concentration at that temperature within a certain heating period. However, extended heat treatment can cause denaturation and cleavage of small soluble oligopeptides, thereby reducing their content. As shown in Fig. 1c, when the boiling temperature and time are held constant, the content of oligopeptides initially increases and then decreases as the solid-to-liquid ratio increases. The oligopeptide content reaches its maximum at a solid-to-liquid ratio of 1:2. Altering the feed-to-liquid ratio significantly affects the oligopeptide content in chicken soup. As the feed-to-liquid ratio increases, the proportion of chicken in the total mass decreases, resulting in fewer soluble small-molecule peptides dissolving into the soup during cooking. This may be because the peptides are fully dissolved at a 1:2 ratio. Beyond this point, increasing the water proportion dilutes the peptides. Therefore, a 1:2 solid-to-liquid ratio is selected as the optimal material-to-liquid ratio.

Based on the single-factor test, the test protocol and results are presented in Table S2. The experimental data were analyzed using multiple regression fitting, resulting in the following fitting equation:

Y = 1.52 + 0.330 A-0.114B + 0.038C-0.140AB + 0.104 AC + 0.092 BC-1.38A^2^–0.264B^2^–0.354C^2^。.

To evaluate the validity of the regression equation, an analysis of variance (ANOVA) was performed on the experimental results using Design-Expert 13. As shown in Table S3, the adjusted coefficient of determination (R^2^_adj_) is 0.9886, indicating that the model explains 98.86 % of the variability in the response variable. The model's *p*-value is less than 0.0001, while the p-value for lack of fit is 0.0555. These results demonstrate that the model is highly accurate and reliable, making it suitable for analyzing and predicting the optimal conditions of the extraction process (Chen et al., 2024). By establishing a three-dimensional response surface and a two-dimensional contour map, the effects of extraction time, extraction temperature, and solid-liquid ratio on the oligopeptide content in chicken soup were analyzed to determine the optimal extraction conditions (Fig. 1d-i). The results show that extraction temperature has the most significant impact on oligopeptide content, followed by extraction time, and finally the solid-liquid ratio. Among the interactions studied, the combination of extraction time and solid-liquid ratio had the greatest effect. The optimal conditions identified were an extraction temperature of 90.87 °C, an extraction time of 3.77 h, and a solid-liquid ratio of 1:2.04. Under these conditions, the model predicted an oligopeptide content of 1.552 mg/mL. For practical application, these conditions were adjusted to an extraction temperature of 90 °C, an extraction time of 4 h, and a solid-liquid ratio of 1:2. Under these adjusted conditions, the oligopeptide content measured was 1.554 mg/mL, closely matching the model's prediction. This indicates that the model effectively captured the preparation process of oligopeptides and accurately predicted their content.

### 3.2 Analysis of isolation and purification of peptides

**Fig. 2:**
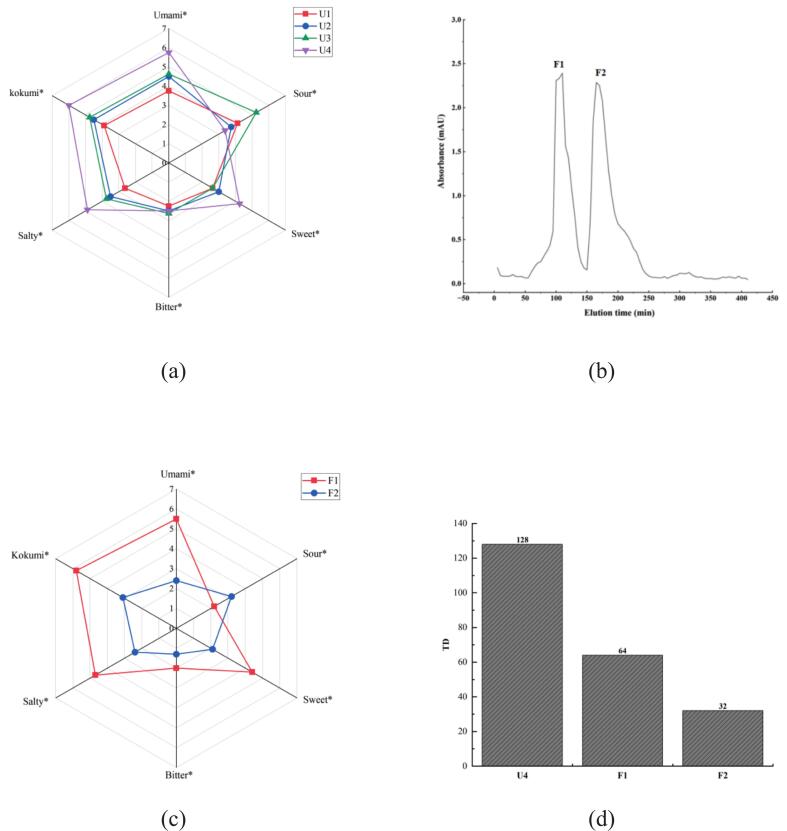
**Sensory evaluation of UF components in Wuding chicken soup.** Radar chart of sensory evaluation results of UF components (a); Gel filtration chromatogram of the U4 fraction with higher kokumi intensity from UF (b); Sensory evaluation of fractions after GFC (c); TD values of UF and GFC components in Wuding chicken soup (d); the * expression significant differences in the respective attributes (*P* < 0.05).

According to molecular weight, ultrafiltration of Wuding chicken soup yielded four distinct fractions: U1 (Mw > 5000 Da), U2 (3000 Da < Mw < 5000 Da), U3 (1000 Da < Mw < 3000 Da), and U4 (Mw < 1000 Da). The lyophilized fractions underwent descriptive evaluation after being reconstituted in chicken broth at a concentration of 1 mg/mL for sensory assessment. As illustrated in Fig. 2a, the U4 fraction demonstrated the highest kokumi intensity, followed by U3 and U2, while U1 showed the lowest kokumi scores. Quantitative sensory analysis revealed strong taste scores of 3.88, 4.50, 4.75, and 6.00 for U1, U2, U3, and U4, respectively. Notably, the U4 fraction exhibited a complex flavor profile dominated by umami, sweet, salty, and kokumi characteristics. These findings suggest that kokumi-active peptides in chicken soup are primarily concentrated in the low molecular weight fraction (<1000 Da). This outcome is in line with earlier research that found the highest kokumi sensory ratings are consistently attained by fractions produced by ultrafiltration in the 0–1000 Da range (Li et al., 2023; Yan et al., 2021; Yang et al., 2022). These findings prompted us to further purify and detect the <1000 Da portion of Wuding chicken soup.

Using Sephadex G-15 gel filtration chromatography, the U4 fraction was effectively divided into two separate components (F1 and F2), as illustrated in Fig. 2b. Two distinct peaks were visible in the chromatographic profile: peak F1 eluted between 50 and 150 min, while peak F2 appeared between 150 and 250 min. Both peaks had elution times of one hundred minutes. The separation between these components was achieved with good resolution. After collection and lyophilization, each component was reconstituted in chicken broth at a concentration of 1 mg/mL for descriptive sensory evaluation. As depicted in Fig. 2c and Fig. 2d show that the two components, F1 and F2, mainly show the characteristics of kokumi and umami but also have a certain salty and sweet taste. This shows that kokumi peptide has strong flavor characteristics but also brings a variety of different flavors, such as umami, salty, and sweet tastes. This discovery tells us that kokumi peptide may have a more complex effect on taste and can change several taste sensations at the same time. During processing, F1's kokumi score ranks after U4, with a taste dilution value of 64. Another point is that F1 performs better than F2 in terms of saltiness, umami, and sweetness (*P* < 0.05). Taken together, the results of gel-filtered chromatography show that F1 has the strongest kokumi characteristic, which may be because the component contains more kokumi-active substances. Consequently, F1 was chosen for further amino acid sequence analysis.

### 3.3 Analysis of identification of peptides

To characterize the kokumi peptides, the amino acid sequences of peptides in gel fraction F1 was determined by Nano-HPLC-MS/MS. Table 1 and Fig. S1 of the supplemental material contain comprehensive details and secondary mass spectra of the fifteen discovered peptides. Protein sequence number, reliability score, chromatographic peak area, and amino acid count were used to figure out the peptides. The fifteen peptides identified include TPVPAS (TS-6), SYTPVPA (SA-7), YTPVPASA (YA-8), TPVPASASY (TY-9), SYTPVPASA (SA-9), TPVPASASYG (TG-10), SYTPVPASAS (SS-10), RIAVPAPR (RR-8), GHFRAAVPS (GS-9), DERIAVPA (DA-8), DERIAVPAK (DK-9), LPVPAFN (LN-7), VPAFNVING (VG-9), SAAPAAPVPA (SA-10), and AGFAGDDAPR (AR-10), with peptide lengths ranging from 6 to 10 amino acids. While previous studies have shown that kokumi peptides are typically small, consisting of 2–5 amino acids without specific structural motifs, recent evidence indicates that longer-chain peptides may exhibit more pronounced kokumi-enhancing effects. Longer peptides can greatly improve the qualities of kokumi, as demonstrated by the improved thickness and continuity of chicken broth supplemented with the peptides RGENESEEEGAIVT and TESSSE (Zhang, Zhao, et al., 2019). Notably, the longest kokumi peptide discovered to date is RGENESEEEGAIVT, which is derived from peanut protein isolate hydrolysates. The protein hydrolysates of chicken and puffer fish (*Takifugu obscurus*) were also used to extract additional longer-chain kokumi peptides, including RWDGRG, KGRYER, YKCKDXXLR, WVNEEDHL, NSLEGEFKG, and KDLFDPVIQD (Zhang, Ma, et al., 2019). Given that longer-chain peptides may have better functional qualities than shorter peptides, these results highlight the need to broaden the scope of research on kokumi compounds.

**Table 1:** Fifteen peptides were identified and screened from F1 components.

Number	Peptides	Molecular weight	Length	m/z	Retention time	-10lgP
1	TPVPAS	570.3013	6	571.3103	9.44	22.45
2	SYTPVPA	733.3646	7	734.3738	21.85	23.82
3	YTPVPASA	804.4017	8	805.411	20.14	32.35
4	TPVPASASY	891.4338	9	892.4423	23.2	40.49
5	SYTPVPASA	891.4338	9	892.4436	24.13	35.02
6	TPVPASASYG	948.4552	10	949.4657	20.9	44.97
7	SYTPVPASAS	978.4658	10	979.4749	21.29	32.74
8	RIAVPAPR	878.545	8	293.8559	13.75	20.45
9	GHFRAAVPS	940.4879	9	471.252	11.31	20.76
10	DERIAVPA	869.4606	8	870.4707	24.5	30.4
11	DERIAVPAK	997.5556	9	333.5267	13.91	30.83
12	LPVPAFN	756.417	7	757.4275	52.05	36.01
13	VPAFNVING	929.4971	9	930.5056	51.9	33.93
14	SAAPAAPVPA	850.4548	10	851.465	22.36	32.53
15	AGFAGDDAPR	975.441	10	488.7294	12.12	30.77

### 3.4 Analysis of kokumi activity and toxicity prediction of peptides

To acquire more about the kokumi activity and flavor properties of the discovered peptides, four peptides were selected for detailed analysis using the BIOPEP database (Iwaniak et al., 2016). The kokumi potential of these peptides was evaluated using the PeptideRanker predictor (Wang et al., 2023), with detailed results provided in Table S4 of the supplementary material. There are four peptides selected in the study, one is a heptapeptide, one is a nonapeptide, and the remaining two are decapeptides, and their molecular weight is between 756.417 and 975.441 Da. In order to determine whether these four peptides can be used in food or medicine, we have done a comprehensive test of their safety and tested various functional properties (Wang et al., 2023). Through ToxinPred, key characteristics such as toxicity, hydropathicity, hydrophobicity, hydrophilicity, and steric hindrance were predicted. Detailed results can be found in Table S4 of the supplementary material. The analysis revealed that all four peptides have a PeptideRanker scores above 0.5, indicating significant biological activity and a non-toxic profile. These favorable properties confirm their potential for safe use in food and health-related products, making them suitable candidates for subsequent experimental research.

### 3.5 Analysis of molecular docking

Ohsu et al. (2010) discovered that the kokumi taste receptor, known as calcium-sensing receptor (CaSR), belongs to Group C of the G protein-coupled receptor (GPCR) family, along with the umami receptor (T1R1 + T1R3) and sweet receptor (T1R2 + T1R3). The CaSR consists of 1078 amino acids and is located in the surface membrane of cells. It is not only expressed in type II and type III taste cells of taste buds at the back of the tongue, but also in numerous other organs, including the kidney, liver, heart, lung, gastrointestinal tract, pancreas, parathyroid gland, and central nervous system, and participates in a series of biological functions (Conigrave, 2016). Yusuke et al. (2016) and Kuroda et al. (2014) further investigated the relationship between the sensory evaluation effect of kokumi taste peptide and the perceptual activity of CaSR. The results showed that there was a significant positive correlation between the taste effect of kokumi taste peptide and the perception signal of the CaSR, indicating that CaSR is the key receptor for the human body to perceive the kokumi taste.

**Fig. 3:**
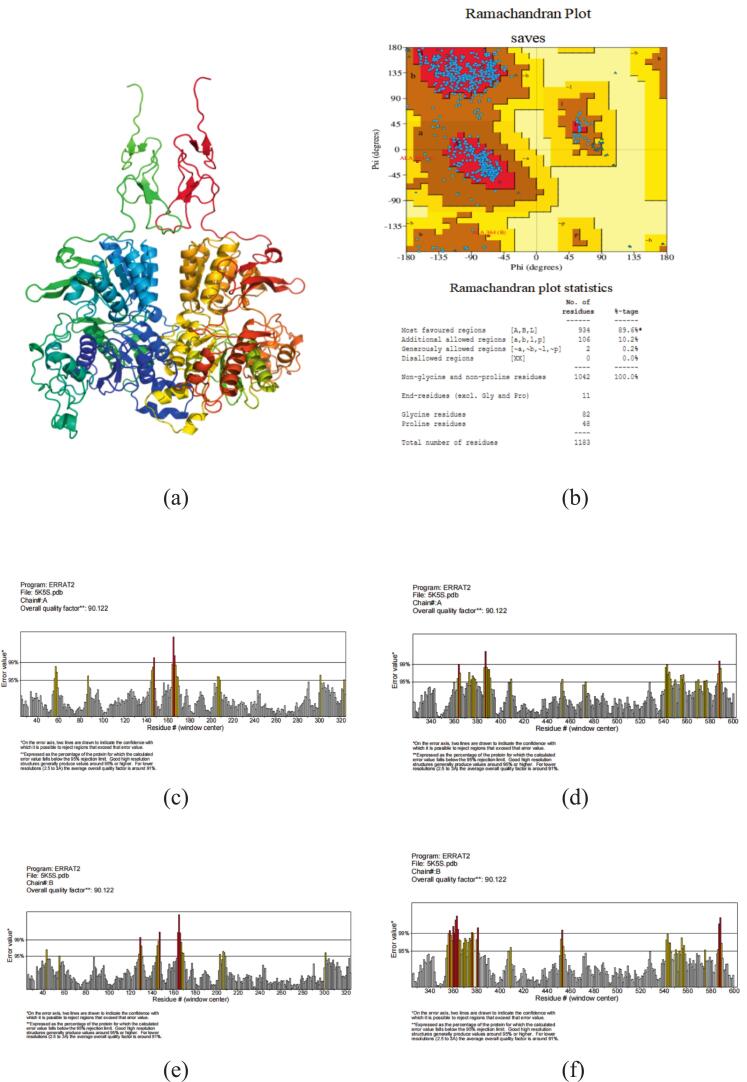
**The homology model structure of the kokumi receptor and its reliability validation.** (a) The homology model structure of the kokumi receptor with the A chain of CaSR on the left and the B chain of CaSR on the right; (b) Ramachandran plot of the model; (c-f) The overall ERRAT quality factor of the homology model.

One effective method for seeing where proteins interact to tiny molecules is molecular docking. Prior to docking, a CaSR homology model was built and its dependability confirmed. In Fig. 3a, the homology model is displayed. With the A chain of CaSR on the left and the B chain of CaSR on the right. The homology model's dependability was demonstrated by the Raman map (Fig. 3b), which revealed that 99.8 % (>90 %) of the amino acid residues were in the acceptable region (89.6 % were in the most preferred regions, and 10.2 % were in extra allowed regions), while 0.2 % were in the prohibited region (Chang et al., 2023; Deng et al., 2021). As shown in Fig. 3(c-f), the overall ERRAT quality factor of the model was 90.12 %, which means that the calculation error is below the 99 % rejection limit of 90.12 % residues. In summary, these data confirm that the homology model is reliable and can be used for molecular docking.

**Fig. 4:**
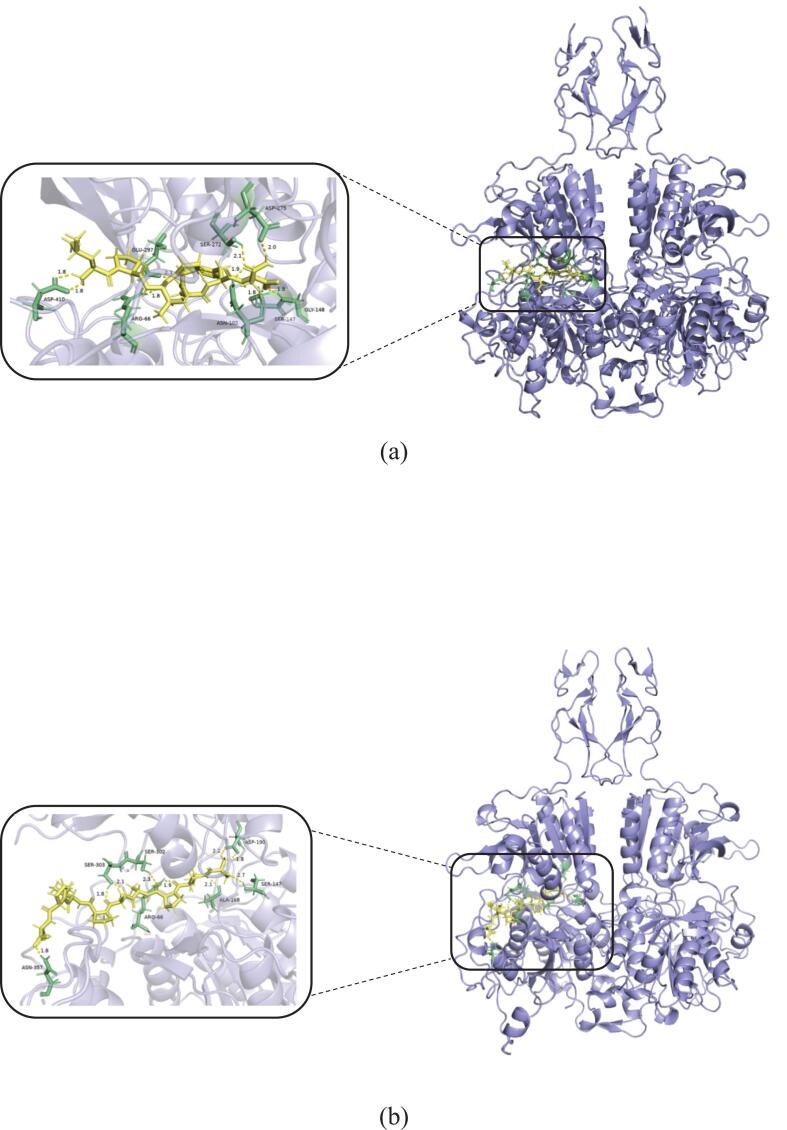

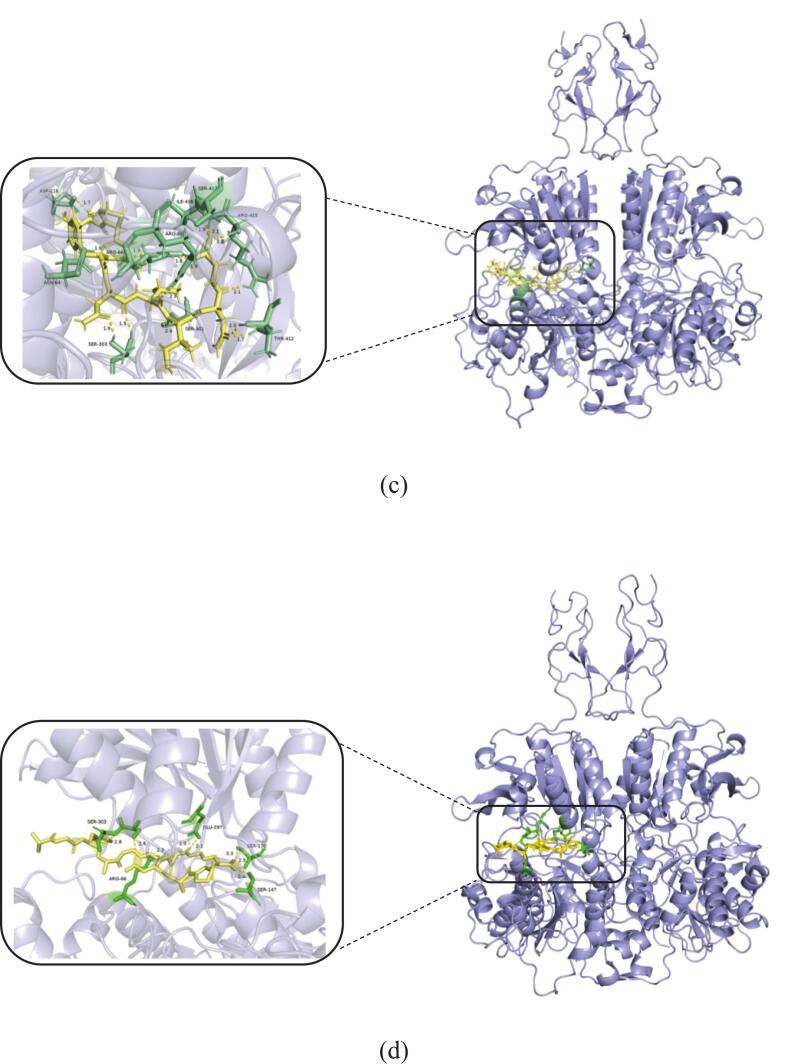
**Molecular docking results of kokumi peptides and partial enlarged detail of hydrogen bond interactions.** (a, LN-7; b, SA-10; c, VG-9; d, AR-10) with kokumi receptor CaSR.

AlphaFold v2.3 software was used to simulate the molecular docking between the kokumi peptide sequences and the receptor. PyMOL and Schrodinger tools were then used for visual analysis. The scores of pTM and ipTM are both derived from TM score, which are used to gauge the accuracy of the entire structure of the docking results. For short chains or small structures, the TM score is extremely stringent (Zhang & Skolnick, 2004). A pTM score higher than 0.5 means that the overall predicted folding of the composite may be similar to the real structure. Accuracy of ipTM in measuring the predicted relative position of subunits in the complex. A value greater than 0.8 indicates a confident, high-quality prediction, while a value lower than 0.6 indicates that the prediction may fail. The ipTM value between 0.6 and 0.8 is a gray area, and the prediction may be correct or incorrect. PTM, ipTM, and comprehensive score are all in the range of 0–1, and the closer to 1, the better. The findings are displayed in Table S5 of the supplemental materials. The pTM score of AR-10 is the highest (0.87), followed by LN-7 (0.86), SA-10 (0.86), and VG-9 (0.86), indicating that the overall predicted folding of the complex formed by CaSR and peptide may be similar to the real structure. Secondly, the ipTM scores of the complexes formed by the four peptides and CaSR are all greater than 0.8, which indicates that the predicted relative position has high-quality accuracy. The structural formulas of the kokumi peptides are illustrated in Fig. S2 of the supplementary material. Molecular docking experiments revealed that all four kokumi peptides formed stable complexes with CaSR, as depicted in Fig. 4. Specifically, the study looked at how peptides bind to the CaSR protein, and we found that these peptides are mainly embedded in the A-chain, which shows that most of their binding sites are in this region. This finding is similar to the previous research done by other scientists, who also believe that the A-chain is the favorite place to combine with kokumi peptides (Feng et al., 2024). But our experiments are not the same as before, because previous studies only stared at the A-chain for docking analysis, and we took into account both chains.

**Fig. 5:**
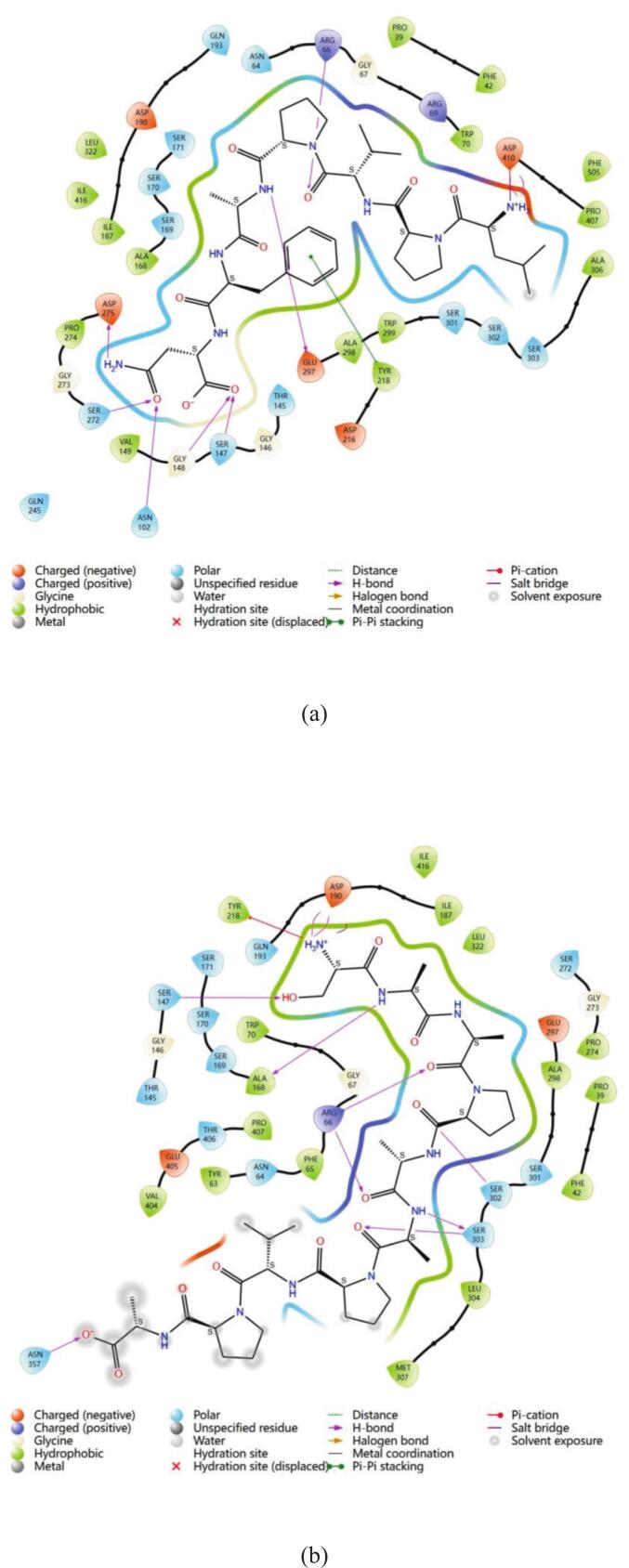

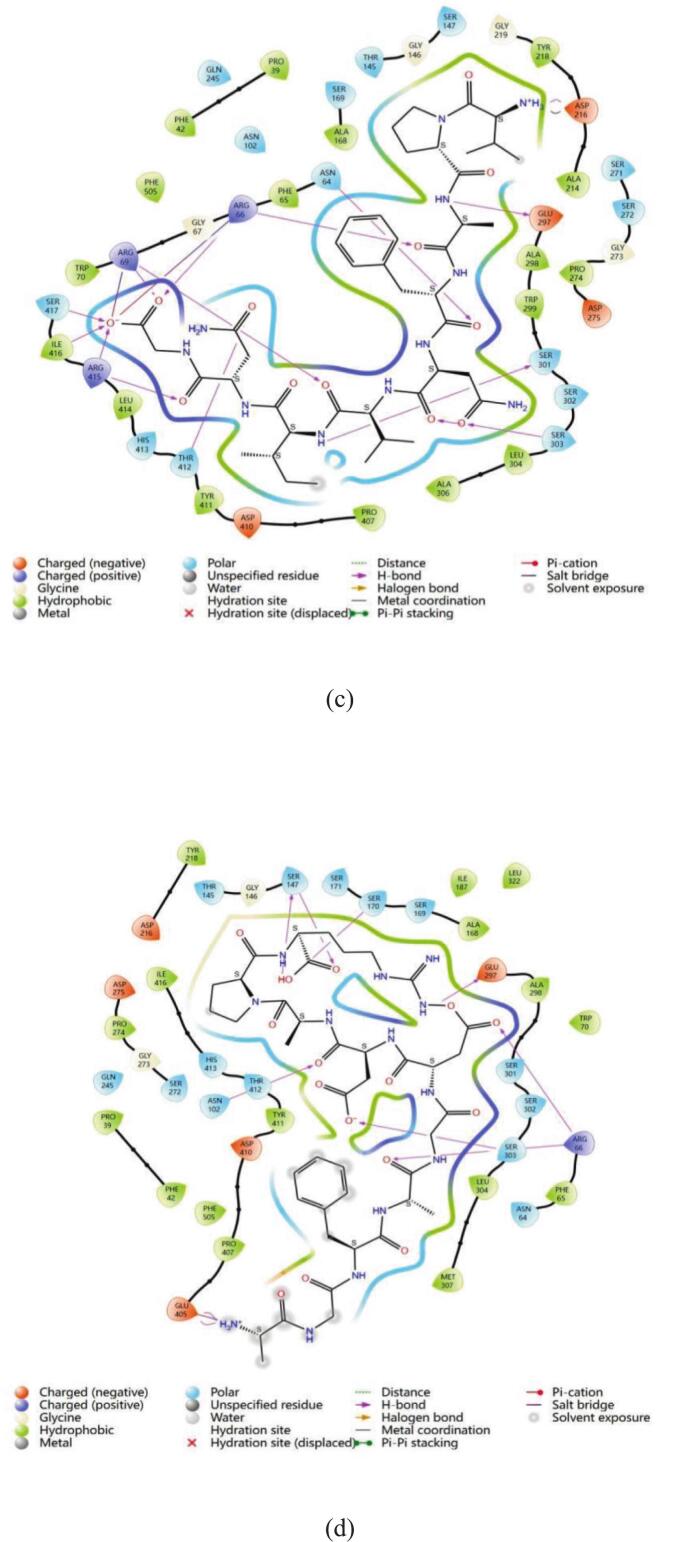
**The interaction network maps of kokumi peptides with the kokumi receptor CaSR.** (a, LN-7; b, SA-10; c, VG-9; d, AR-10).

These four kokumi peptides are primarily linked to the receptor in a number of ways, according to molecular docking experiments. These include hydrophobic contact (alkyl and pi-alkyl), hydrogen bonding (conventional and carbon hydrogen bonds), and electrostatic interactions (salt bridges, attractive charge, and pi-anion). It is evident from the 2D diagram in Fig. 5 how the four kokumi peptides interact with the active residues of CaSR. The associated binding locations are shown in Table 2. All four peptides (LN-7, SA-10, VG-9, and AR-10) contained the amino acid residue Arg 66, which had the most interactions between ligands and receptors. Ser 303, Ser 147, Glu 297, and Arg 69 were next in line. This suggested that important amino acid residues Arg 66, Ser 303, Ser 147, Glu 297, and Arg 69 were essential for the interaction and binding of CaSR with kokumi peptides. The results of this investigation aligned with previous studies. According to Lao et al. (2024), kokumi peptides mainly engage in electrostatic interactions with CaSR through important residues like Asp 275, Asn 102, Pro 274, Trp 70, Tyr 218, and Ser 147. Furthermore, these peptides can interact with CaSR, primarily through the role of polarly dissolved free energy and the van der Waals force. The more important binding sites are Asp 275, Ile 416, Pro 274, and Arg 66, Ala 298, and Tyr 218. From these results, it can be seen that the two amino acid residues of Ser 147 and Arg66 are particularly important, and they play a key role in the interaction between kokumi peptide and CaSR. To further suppor this point, Yamaguchi et al. (2025) conducted structural analysis and revealed the fundamental interaction between kokumi peptide *γ*-EVG and CaSR. Key residues involved in these interactions included Pro 39, Phe 42, Arg 66, Ser 147, and Glu 297. This work further emphasizes the importance of Arg 66, Ser 147, and Glu 297 as crucial amino acid residues that are necessary for the binding and interaction between kokumi peptides and CaSR. Both studies highlight the importance of Arg 66 and Ser 147 in kokumi taste perception by demonstrating how they play a key role in the molecular recognition and binding processes between kokumi peptides and CaSR.

**Table 2:** The binding force between kokumi peptides and key amino acid residues around the active site in CaSR.

Binding sites	LN-7	SA-10	VG-9	AR-10
Glu 297	+		+	+
Glu 405				+
Gly 148	+			
Asp 275	+			
Asp 410	+			
Tyr 218	+	+		
Ser 147	+	+		++
Ser 170				+
Ser 272	+			
Ser 303		++	++	++
Ser 302		+		
Ser 301			+	
Ser 417			+	
Arg 66	+	++	+++	++
Arg 69			+++	
Arg 415			++	
Thr 412			+	+
Ile 416			+	
Ala 168		+		
Asn 64			+	
Asn 102	+			+
Asn 357		+		

Note: “+” indicates the number of interactions between the ligand and receptor, and a.

“+” indicates an interaction force.

To better understand the hydrogen bonding interactions between kokumi peptides and CaSR, Fig. 4 and Table S6 of the supplemental material show the hydrogen bonding forces and related data. In the figures, the yellow dotted lines represent hydrogen bonds, yellow denotes the kokumi peptides, and green indicates the amino acid residues involved in hydrogen bonding with the peptides. LN-7 formed 9 hydrogen bonds with CaSR, involving receptor residues Asp 410, Arg 66, Glu 297, Ser 272, Asp 275, Asn 102, Gly 148, and Ser 147. SA-10 also formed 9 hydrogen bonds with CaSR, interacting with residues Asn 357, Ser 303, Ser 302, Arg 66, Ala 168, Ser 147, and Asp 190. VG-9 established 15 hydrogen bonds with CaSR, primarily involving residues Thr 412, Ser 301, Ser 303, Arg 415, Ile 416, Ser 417, Asp 216, Arg 66, Asn 64, and Arg 69. AR-10 formed 11 hydrogen bonds with the CaSR, interacting with residues Glu 405, Ser 303, Arg 66, Glu 297, Asn 102, Ser 147, and Ser 170. Arg 66 formed hydrogen bonds with all four peptides. These peptides included LN-7, SA-10, VG-9, and AR-10. Ser147 showed binding activity with three peptides. The affected peptides were LN-7, SA-10, and AR-10. Only three hydrogen bonds measured between 2.5 Å and 2.7 Å. Most bonds ranged from 1.7 Å to 2.3 Å. These findings confirm kokumi peptides stabilize receptor complexes. Their binding relies on hydrogen bonds and hydrophobic forces. This mechanism aligns with prior research on CaSR interactions. Feng et al. (2024) analyzed Agaricus bisporus peptides. Their docking simulations revealed three interaction types. Hydrogen bonds, electrostatic forces, and hydrophobic contacts all contributed. This supports our observed binding patterns.

## 4 Conclusion

In this work, the extraction conditions of oligopeptides in Wuding chicken soup were determined through process optimization and kokumi peptides from Wuding chicken soup were isolated, purified, identified, and screened using UF, GFC, and Nano-HPLC-MS/MS. Fifteen kokumi peptides (TS-6, SA-7, YA-8, TY-9, SA-9, TG-10, SS-10, RR-8, GS-9, DA-8, DK-9, LN-7, VG-9, SA-10, and AR-10) were identified. Four peptides were selected based on their predicted kokumi activity characteristics, and their water solubility and toxicity were tested. The results revealed that LN-7 exhibited the highest activity score, followed by AR-10, SA-10, and VG-9. Homology modeling and molecular docking analyses suggested that Arg 66, Ser 303, Ser 147, Glu 297, and Arg 69 are likely the key active sites mediating the interaction between kokumi peptides and CaSR. Additionally, hydrogen bonding and hydrophobic interactions were identified as crucial forces in the binding of peptides LN-7, SA-10, VG-9, and AR-10 to the CaSR. This research successfully identified four novel kokumi peptides from Wuding chicken soup, clarified the mechanisms underlying their flavor and offered insightful information for the theoretical investigation and screening of kokumi peptides. To create a strong basis for the industrial use of kokumi peptides, it is imperative to investigate safe and effective bioengineering techniques in order to generate target flavor peptides at a low cost and high yield.

## CRediT authorship contribution statement

**Chunfang Yang:** Writing – original draft, Visualization, Software, Investigation, Data curation. **Yanfei Du:** Software, Investigation, Data curation, Conceptualization. **Guiying Wang:** Writing – review & editing, Visualization, Project administration, Funding acquisition. **Jia Liu:** Supervision, Software. **Jiayan Tan:** Methodology, Formal analysis. **Shuai Tang:** Methodology, Data curation, Conceptualization. **Yuan Liu:** Software, Resources, Investigation. **Hongbin Pan:** Supervision, Resources, Methodology. **Guozhou Liao:** Writing – review & editing, Validation, Supervision, Funding acquisition.

## Institutional review board statement

The Human Ethics Committee of Yunnan Agricultural University does not have a practice of approving sensory experiments on food. Therefore, this experiment was performed in accordance with the Declaration of Helsinki and informed consent was obtained from all assessors.

## Declaration of competing interest

The authors declare that they have no known competing financial interests or personal relationships that could have appeared to influence the work reported in this paper.

## Data Availability

I have shared the link to my data at the Attach File step.

## Appendix A Supplementary data

Supplementary material Image 1
