# Supplementary material for: Discovery and mechanistic profiling of novel kokumi peptides from Wuding chicken soup
Source: Food Chem X. 2025 Oct 14;31:103171. doi: 10.1016/j.fochx.2025.103171 (PMC12554036; doi:10.1016/j.fochx.2025.103171)
Supplement: Supplementary file 1 — Supplementary material [file mmc1.docx]

**Table S1** Response surface test factors and levels.

| Factors | Levels | | |
| --- | --- | --- | --- |
|  | -1 | 0 | 1 |
| A Extraction temperature (℃) | 85 | 90 | 95 |
| B Extraction time (h) | 3 | 4 | 5 |
| C Solid-liquid ratio | 1:1 | 1:2 | 1:3 |

**Table S2** Response surface experimental design and results table.

| Run | A  Extraction temperature (℃) | B  Extraction time (h) | C  Solid-liquid ratio | Y  Oligopeptide content (mg/mL) |
| --- | --- | --- | --- | --- |
| 1 | 85 | 5 | 1:2 | 0.734 |
| 2 | 90 | 4 | 1:2 | 1.514 |
| 3 | 85 | 4 | 1:3 | 0.601 |
| 4 | 85 | 3 | 1:2 | 0.794 |
| 5 | 95 | 4 | 1:1 | 0.934 |
| 6 | 95 | 4 | 1:3 | 1.074 |
| 7 | 85 | 4 | 1:1 | 0.670 |
| 8 | 90 | 4 | 1:2 | 1.488 |
| 9 | 95 | 5 | 1:2 | 0.885 |
| 10 | 90 | 4 | 1:2 | 1.544 |
| 11 | 90 | 3 | 1:1 | 1.063 |
| 12 | 90 | 4 | 1:2 | 1.524 |
| 13 | 90 | 5 | 1:3 | 0.924 |
| 14 | 90 | 4 | 1:2 | 1.524 |
| 15 | 90 | 5 | 1:1 | 0.624 |
| 16 | 95 | 3 | 1:2 | 1.225 |
| 17 | 90 | 3 | 1:3 | 0.994 |

**Table S3** Analysis of variance in the response surface analysis.

| Source | Sum of squares | df | Mean square | F-value | p-value |  |
| --- | --- | --- | --- | --- | --- | --- |
| Model | 1.870 | 9 | 0.208 | 155.65 | <0.0001 | Significant |
| A-Extraction temperature | 0.217 | 1 | 0.217 | 162.83 | <0.0001 | ******* |
| B-Extraction time | 0.103 | 1 | 0.103 | 77.33 | <0.0001 | ******* |
| C-Solid-liquid ratio | 0.011 | 1 | 0.011 | 8.54 | 0.0223 | ***** |
| AB | 0.020 | 1 | 0.020 | 14.68 | 0.0065 | ****** |
| AC | 0.011 | 1 | 0.011 | 8.18 | 0.0244 | ***** |
| BC | 0.034 | 1 | 0.034 | 25.49 | 0.0015 | ****** |
| A^2^ | 0.502 | 1 | 0.502 | 376.11 | <0.0001 | ******* |
| B^2^ | 0.293 | 1 | 0.293 | 219.56 | <0.0001 | ******* |
| C^2^ | 0.527 | 1 | 0.527 | 394.29 | <0.0001 | ******* |
| Residual | 0.002 | 7 | 0.001 |  |  |  |
| Lack of fit | 0.008 | 3 | 0.003 | 6.17 | 0.0555 | Not significant |
| Pure error | 0.002 | 4 | 0.000 |  |  |  |
| Cor total | 1.882 | 16 |  |  |  |  |
|  | R^2^=0.995 | R_adj_^2^=0.9886 | | R_pre_^2^=0.9332 | |  |

Note: * indicates significant difference, *P* < 0.05, ** indicates highly significant difference, *P* < 0.01, and *** indicates highly significant difference, *P* < 0.0001.

**Table S4** Activity and toxicity prediction of kokumi peptides in of Wuding chicken soup.

| Peptides | Ranker | Toxicity | Hydrophobicity | Hydrophilicity | Hydropathicity | Steric hindrance |
| --- | --- | --- | --- | --- | --- | --- |
| LN-7 | 0.62 | Non-Toxin | 0.16 | -0.87 | 0.84 | 0.56 |
| VG-9 | 0.50 | Non-Toxin | 0.16 | -0.82 | 0.94 | 0.65 |
| SA-10 | 0.53 | Non-Toxin | 0.13 | -0.37 | 0.76 | 0.49 |
| AR-10 | 0.56 | Non-Toxin | -0.16 | 0.50 | -0.57 | 0.62 |

**Table S5** Scoring results of kokumi peptides with the kokumi receptor CaSR.

| Peptide | pTM | ipTM | Comprehensive scoring |
| --- | --- | --- | --- |
| AR-10 | 0.87 | 0.85 | 0.92 |
| LN-7 | 0.86 | 0.84 | 0.90 |
| SA-10 | 0.86 | 0.84 | 0.90 |
| VG-9 | 0.86 | 0.83 | 0.90 |

**Table S6** Hydrogen bonding information of kokumi peptides docked with the kokumi receptor CaSR.

| Ligands | Hydrogen bonds number | Hydrogen bond distance(Å) |
| --- | --- | --- |
| LN-7 | 9 | Asp 410:1.8,1.8;Arg 66:1.8;Glu 297:2.1;Ser 272:2.1;  Asp 275:2.0;Asn 102:1.9;Gly 148:1.8;Ser 147:1.8 |
| SA-10 | 9 | Asn 357:1.8;Ser 303:1.8,2.1;Ser 302:2.3;Arg 66:1.9;  Ala 168:2.1;Ser 147:2.7;Asp 190:2.2,1.8 |
| VG-9 | 15 | Thr 412:1.7,2.0;Ser 301:2.6;Ser 303:1.9,1.9;  Arg 415:2.1,1.8;Ile 416:2.1;Ser 417:1.9;Asp 216:1.7;  Arg 66:1.8,1.9;Asn 64:1.9;Arg 69:2.3,1.8; |
| AR-10 | 11 | Glu 405:1.8,1.8;Ser 303:2.5;Arg 66:1.8,1.9,2.2;  Glu 297:1.7;Asn 102:1.9;Ser 147:1.8,1.8;Ser 170:2.1 |


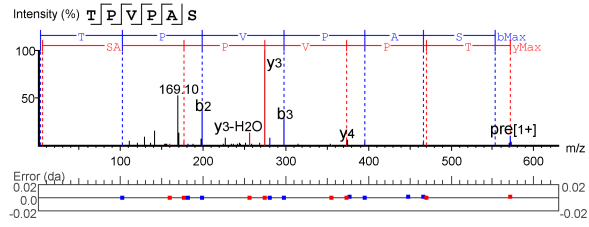

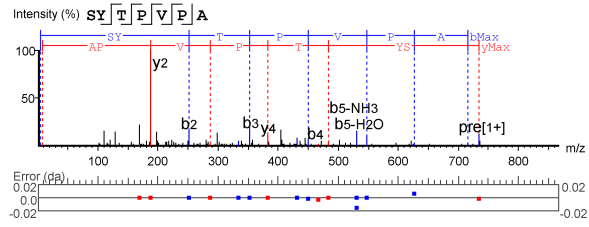


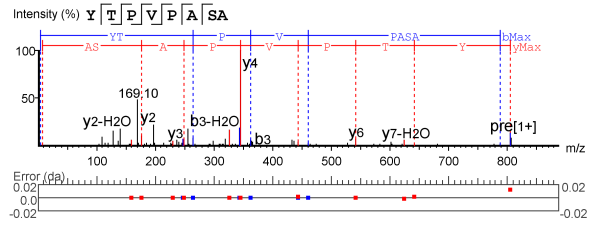

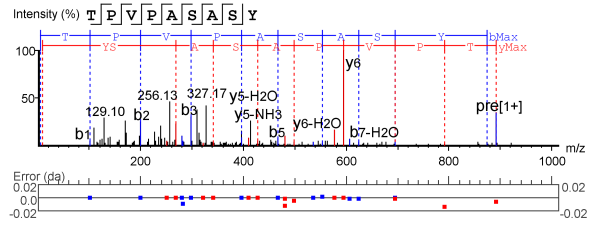


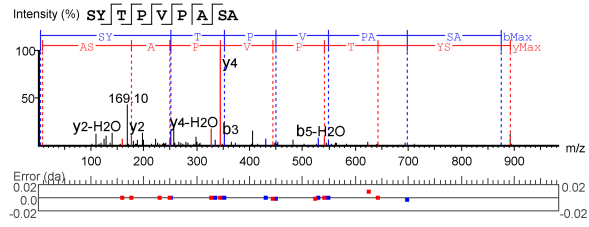

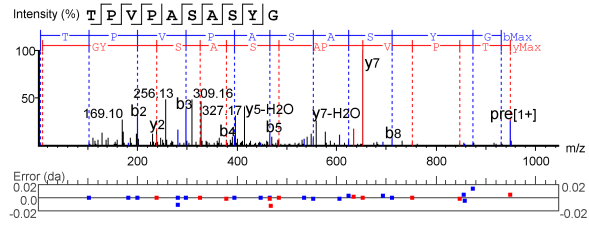


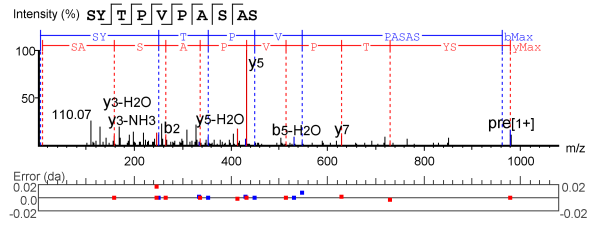

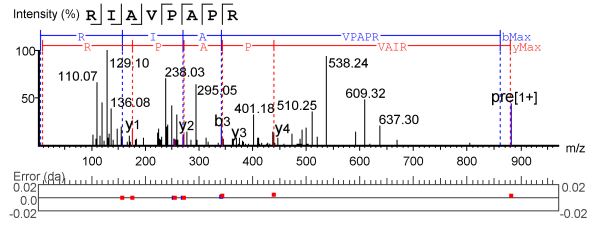


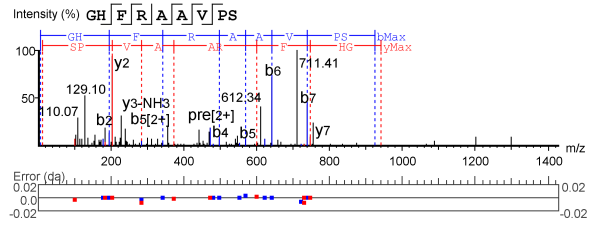

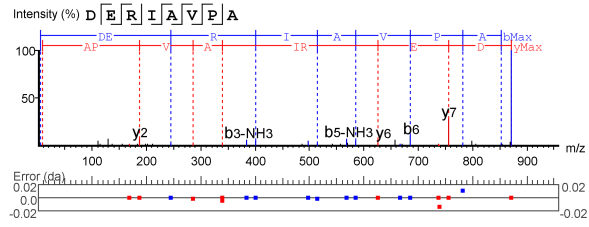


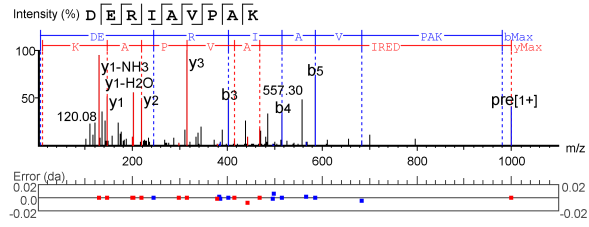

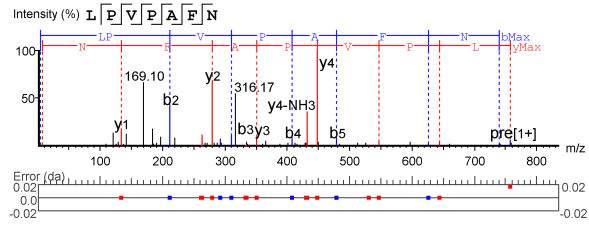


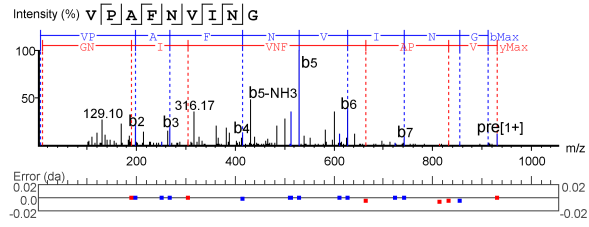

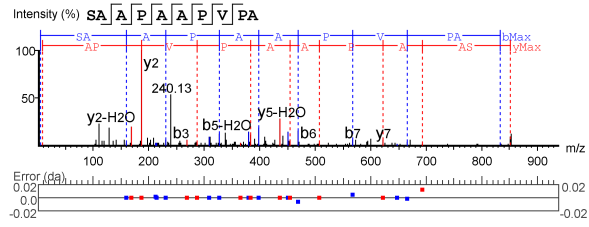


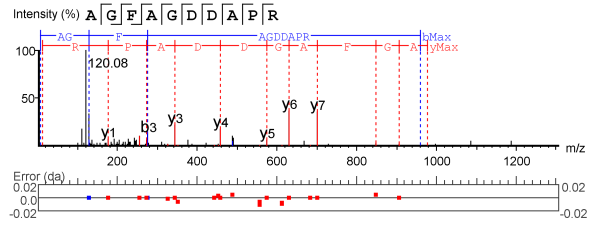


Fig. S1. MS/MS spectrum of fifteen peptides identified by Nano-HPLC-MS/MS in Wuding chicken soup


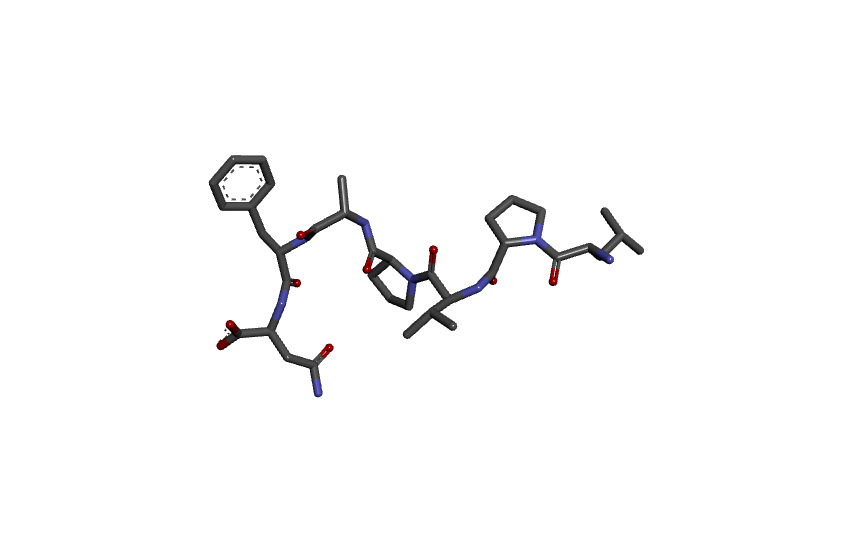

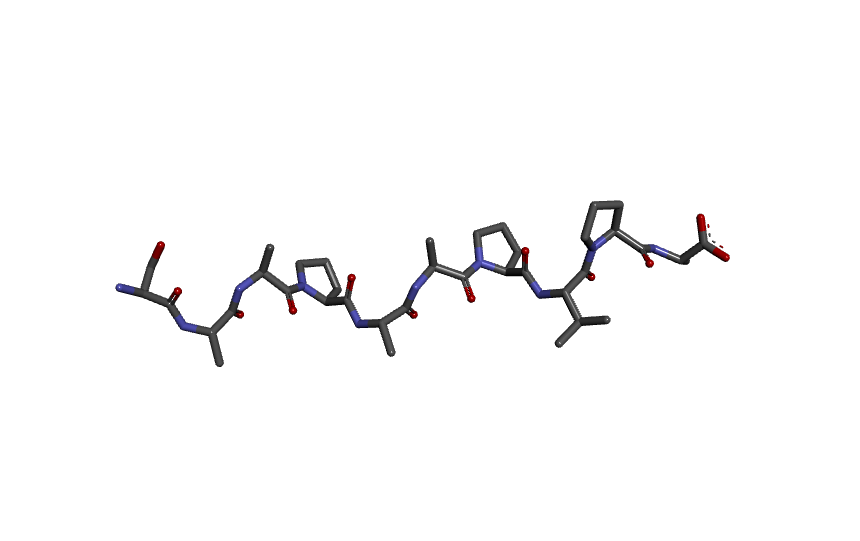


(a) (b)


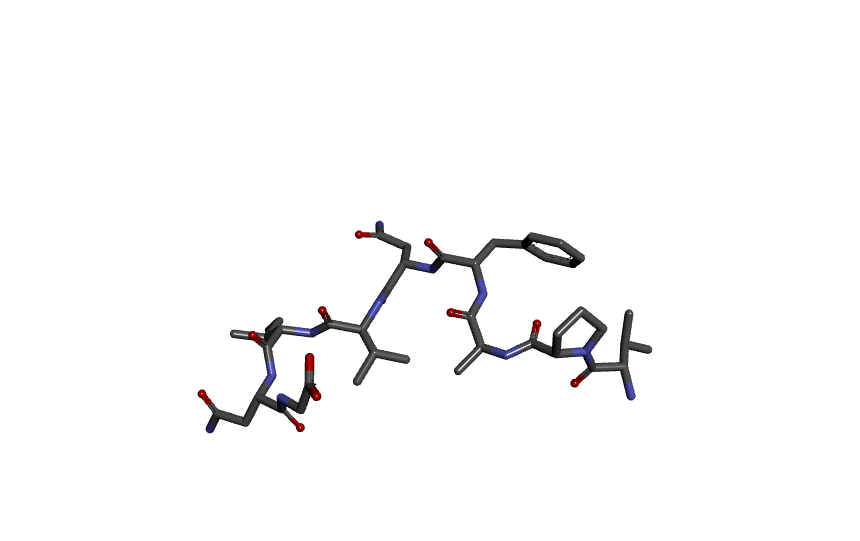

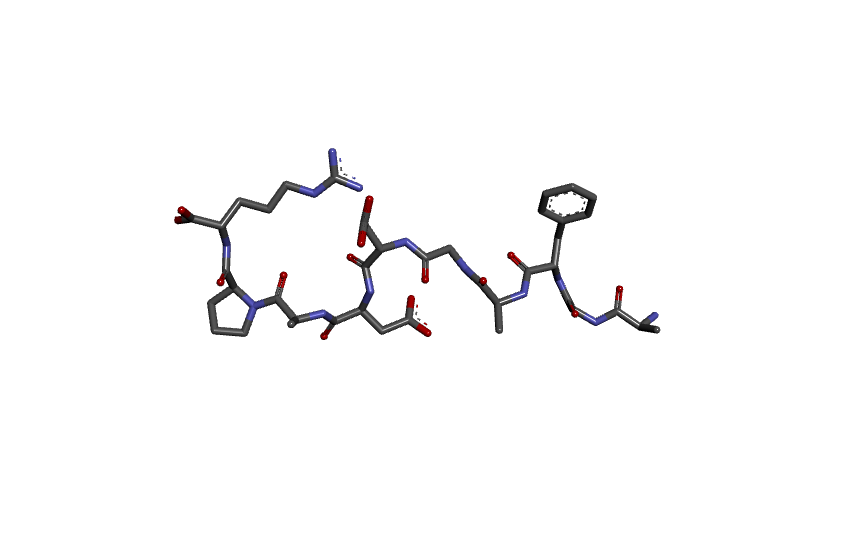


(c) (d)

Fig. S2. Structural formula of four kokumi peptides (a, LN-7; b, SA-10; c, VG-9; d, AR-10).
